# Acute symptoms of depression and traumatic stress in men and women who terminate pregnancy after the detection of fetal anomaly: A prospective observational study

**DOI:** 10.1111/1471-0528.17884

**Published:** 2024-06-20

**Authors:** Mona Bekkhus, Aurora Oftedal, Guttorm Haugen, Berit Mortensen, Anne Kaasen

**Affiliations:** ^1^ Department of Psychology, PROMENTA Research Centre University of Oslo Oslo Norway; ^2^ Division of Mental and Physical Health, Department of Children and Families Norwegian Institute of Public Health Oslo Norway; ^3^ Division of Obstetrics and Gynaecology Oslo University Hospital Oslo Norway; ^4^ Institute of Clinical Medicine University of Oslo Oslo Norway; ^5^ Faculty of Health Sciences Oslo Metropolitan University Oslo Norway

**Keywords:** depressive symptoms, detection of fetal anomalies, termination of pregnancy, traumatic stress

## Abstract

**Objective:**

To assess acute and long‐term stress in men and women after the detection of fetal anomalies leading to pregnancy termination.

**Design:**

Prospective observational study.

**Setting:**

Tertiary referral centre for fetal medicine.

**Population:**

From the initial sample of 180 pregnant women with a fetal anomaly detected by ultrasound examination, a total of 87 women terminated their pregnancy, with 72 partners included in the sample. At the time of detection, the group of women (*n* = 93) and their partners (*n* = 81) who did not terminate the pregnancy following a diagnosis were included as a comparison group.

**Methods:**

These women and their partners were asked to complete the Edinburgh Postnatal Depression Scale (EPDS) and the Impact of Events Scale (IES) questionnaires, both at the time of initial detection and at 6 weeks after the termination of the pregnancy.

**Main outcome measures:**

Responses to the EPDS and the IES at the time of initial detection and at 6 weeks after pregnancy termination.

**Results:**

Women who underwent pregnancy termination reported higher symptom levels of depression, but not traumatic stress, prior to the termination than women who chose not to terminate their pregnancy. Among men, there was a difference across depression and all subscales of traumatic stress (e.g. IES intrusion: mean difference 5.31; 95% CI 2.32–8.31). Women experienced more depressive symptoms over time than men (β = 4.33, *P* < 0.001) and higher symptom levels on all subscales of traumatic stress (e.g. IES intrusion: β = 5.27; *P* < 0.001).

**Conclusions:**

Overall, our study underscores the heightened levels of depression and traumatic stress experienced by prospective parents, particularly prior to the decision to terminate a pregnancy following the detection of a fetal anomaly. Although women generally report more pronounced symptoms, it is noteworthy that men also experience considerable traumatic stress during this challenging time.

## 1 INTRODUCTION

The occurrence of fetal anomalies, identified through ultrasound examination in pregnancy, has been estimated to impact approximately 2%–4% of expectant parents.^1^ Previous studies have shown that the severity of the fetal anomaly influences the decision to terminate the pregnancy.^2^ Once the diagnosis has been made, parents might face the challenging decision of whether to continue or terminate a pregnancy; this decision often needs to be made under acute stress, soon after the discovery of a fetal anomaly.^3^.

Terminating a pregnancy in response to the diagnosis of a fetal anomaly requires an active parental decision.^4^ This active involvement places these families in a grey area between miscarriages and elective terminations for personal reasons, often associated with stigma and stress, and placing immense emotional strain on the affected woman and her partner.^5^, ^6^

The weight of deciding whether to continue or terminate a pregnancy not only has profound implications for a woman's emotional health but also deeply affects expectant fathers. In an earlier study using data from the SOFUS study, fathers anticipating the birth of a child with an ultrasound‐detected fetal anomaly showed heightened symptoms of depression and traumatic stress, compared with a control group.^7^ Similarly, research by Korenromp et al. indicated high levels of postnatal stress symptoms in both parents, although men exhibited slightly fewer symptoms than their female counterparts.^8^ Additionally, a recent qualitative study revealed that fathers experienced intense emotional strain throughout the termination process.^9^ These findings emphasise the importance of a better understanding of the impact that medical termination might have on both expectant parents, and highlight the need for emotional support for both parents. Despite acknowledging these effects, most studies mainly focus on women, often neglecting expectant fathers or not including follow‐up data. Much of the current literature relies on retrospective reports and on qualitative interviews.^10^ There are few quantitative studies with a prospective longitudinal approach. To the best of our knowledge almost all quantitative studies have recorded measures after the termination of pregnancy (TOP),^11^ rather than prospectively in two groups; those with TOP and those without.

The objective of the current study is to measure acute symptoms of depression and traumatic stress shortly after the detection of a fetal anomaly among women and their partners who terminate their pregnancy, and compare these responses with expectant parents who did not terminate their pregnancy. We also examined symptoms of depression and traumatic stress at 6 weeks after TOP.

## 2 METHODS

### 2.1 Study design and participants

**FIGURE 1:**
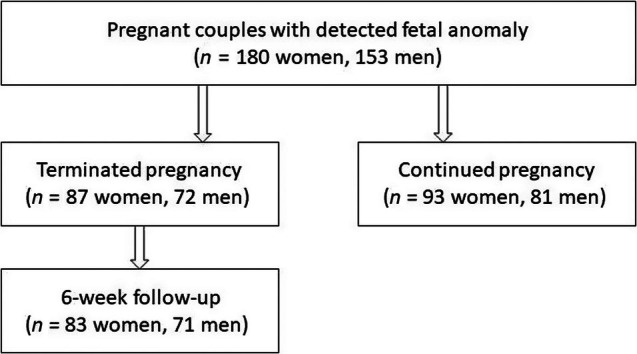
Participant flow chart.

This research is a subsection of a broader, longitudinal investigation, the SOFUS study,^12^ which probes parental stress responses after identifying fetal anomalies by ultrasound examination. The participants were sourced from pregnant women and their partners who were under the care of Oslo University Hospital, Rikshospitalet. The study group was enrolled after a structural fetal anomaly was detected during ultrasound examination in pregnancy. Initially, the sample consisted of 180 expectant mothers and 153 of their partners with a detected fetal anomaly. Of this population, 87 women and their partners (*n* = 72) terminated their pregnancy, and this group was then compared with the group of parents who did not terminate their pregnancy (*n* = 93 women and *n* = 81 partners). Participant flow is shown in Figure 1. All partners in both groups were male.

### 2.2 Procedure

The women were referred to the fetal medicine referral centre at Oslo University Hospital (OUS) Rikshospitalet for an ultrasound examination following suspicions of a fetal abnormality during routine scans at their local hospitals (at around 17–19 weeks of gestation). A fetal medicine specialist conducted the examination and, if needed, additional tests such as genetic or infection tests were performed for inconclusive diagnoses. Consultations with specialists like paediatric neurologists or geneticists occurred when the diagnosis or prognosis was uncertain. Results were continuously shared with the pregnant woman and her partner, who received standard care and follow‐up at the hospital.

The research team, led by a trained midwife, approached the women and their partners for study participation at least 24 h after the ultrasound examination. If they consented, participants were invited to complete questionnaires at the hospital within the next 72 h. No terminations were conducted during this initial period, and all terminations after 12 weeks of gestation require authorisation from the official abortion board. Terminations typically took place about 1 week after the initial data collection (at a median of 19 weeks of gestation plus 1 week), similar to typical terminations for fetal anomalies in Europe,^13^ although the timing could vary owing to diagnostic uncertainties affecting the decision‐making process.^14^

The first set of data was collected within 72 h of detecting the fetal anomaly (T1), with follow‐up data gathered at 6 weeks post‐termination (T2). This study was conducted from May 2006 to February 2009.

### 2.3 Exposure

The exposure variable was termination versus continuation of pregnancy after the detection of fetal anomalies by ultrasound examination.

### 2.4 Main outcome measures

Depression was measured using the Edinburgh Postnatal Depression Scale (EPDS).^15^ The scale consists of ten items that are rated on a Likert scale from 0 ‘not at all’ to 3 ‘most of the time’ (with a total range of 0–30). It has been validated for use during pregnancy,^16^ in the postpartum period,^17^ and for men.^18^ At T1, Cronbach's alpha for the measure was 0.86 for men and 0.88 for women.

Traumatic stress was measured using the Impact of Events Scale (IES),^19^ which is a 22‐item scale that measures psychological reactions to a defined stressful event. In this study, the questions were posed with reference to ‘the child's condition’. The scale consists of three subscales: intrusion (eight items), avoidance (eight items) and arousal (six items). Items were scored on a scale from 0 ‘not at all’ to 5 ‘extremely’, and the scores for items in each subscale were summarised. At T1, Cronbach's alpha for the measure among men was 0.86 for intrusion, 0.79 for avoidance and 0.80 for arousal. Among women, Cronbach's alpha was 0.79 for intrusion, 0.79 for avoidance and 0.75 for arousal.

### 2.5 Covariates

Information regarding the education level, marital status, previous children, previous miscarriages and previous terminations of pregnancy for the participants was collected using self‐report questionnaires at the time of inclusion. The severity of detected anomalies and gestational age at the time of detection was collected using electronic medical charts.

The severity of the detected anomalies was classified according to Kaasen et al.,^12^ in the following four categories:

1. Lethal or serious, with no available treatment (e.g. skeletal dysplasia).
2. Serious, with treatment available (e.g. hypoplastic left heart syndrome or diaphragmatic hernia).
3. Mild to moderate severity, with treatment available (e.g. gastroschisis or duodenal atresia).
4. Classification not available, awaiting clarification. Here, the prognosis is highly dependent on the results of invasive tests (e.g. bilateral clubfoot with chromosomal soft markers), or a reliable diagnosis was not available at inclusion because of an incomplete ultrasound examination.

Furthermore, the detected anomalies were categorised as either ambiguous or not ambiguous. Anomalies were classified as ambiguous for one of two reasons: either the anomaly had significant inherent variation in its prognosis or a definite diagnosis was dependent on the results of further investigations. For example, this may be a condition such as bilateral clubfoot, which is known to be associated with syndromes that may not be apparent prenatally.

### 2.6 Analytical strategy

Acute symptoms of depression and traumatic stress shortly after the detection of fetal anomalies were compared among men and women who did and did not terminate the pregnancy using an independent samples *t*‐test. The analyses were performed using SPSS 28 (IBM, Armonk, NY; USA). To examine symptoms of depression and traumatic stress over time among participants who terminated the pregnancy, linear mixed models were fitted from shortly after the time of diagnosis (prior to pregnancy termination) to 6 weeks after pregnancy termination. The models were calculated using R 4.2.2 (R Foundation for Statistical Computing, Vienna, Austria) with the package Lme4 and *α* = 0.05.^20^ For each outcome variable, four models were created. The null model included only the dependent variable with a random intercept for each family. In model 1, time was added as a fixed effect, and in model 2, time and parent gender were added as fixed effects. In model 3, time and parent gender were entered as fixed effects, along with their interaction effect. Model parameters were estimated by means of maximum likelihood, and competing models were compared using the likelihood ratio test.^21^ The influence of potential confounding variables was examined using multiple regression analysis. Maternal age, education, previous children and the severity of the detected anomaly were entered as predictor variables, and depression, intrusion, avoidance and arousal among parents who terminated the pregnancy were entered as dependent variables one by one. Predictor variables that were significantly related to psychological outcomes were controlled for in the linear mixed models.

### 2.7 Ethics approval

The study was approved by the Regional Committee for Medical Research Ethics, Southern Norway (ref. no. S‐05281). All participants gave their written informed consent prior to participation.

## 3 RESULTS

### 3.1 Descriptive statistics

The sociodemographic characteristics of the participants are shown in Table 1. There were no significant differences among the women and men who terminated or did not terminate the pregnancy in terms of sociodemographic variables at inclusion. However, cases with serious or lethal severity of detected anomalies were higher in the group that terminated the pregnancy than in the group that did not terminate the pregnancy.

**TABLE 1:** Sociodemographic characteristics of the participants.

	Terminated pregnancies(*N* = 87 women, *N* = 72 men)		Continued pregnancies (*N* = 93 women, *N* = 81 men)	
	Women	Men	Women	Men
	*n* (%)	*n* (%)	*n* (%)	*n* (%)
Married or cohabiting	84 (98%)	68 (98%)	90 (97%)	77 (96%)
Education				
High school or less	30 (34%)	38 (53%)	36 (39%)	37 (47%)
University or trade school, up to 4 years	33 (38%)	16 (23%)	31 (34%)	19 (24%)
University, 4 years or more	23 (26%)	17 (24%)	24 (26%)	23 (29%)
Severity of detected anomaly				
Lethal or serious, with no treatment available	42	–	7	–
Serious, with treatment available	27	–	17	–
Moderate to mild severity, with treatment available	4	–	45	–
Classification not available	14	–	24	–
Diagnostic ambiguity				
No ambiguity	34	–	33	–
Ambiguity	53	–	60	–
Gestational age at detection, median weeks (min–max)	19 (12–37)	–	20 (12–38)	–
Previous children	51 (60%)	41 (58%)	46 (49%)	39 (48%)
Previous termination of pregnancy	13 (18%)	–	21 (25%)	–
Previous miscarriage	24 (28%)	–	17 (18%)	–

Overall, at T1 women who terminated the pregnancy had a mean depression score of 13.38 (SD 5.47), and men had a mean depression score of 8.9 (SD 4.87) (Table 2). In the Norwegian populations, a cut‐off score of 10 has been found to be indicative of clinical depression,^22^ thus women who terminated the pregnancy scored higher than the suggested indication score for clinical depression.

**TABLE 2:** Mean scores and standard deviations for depression and traumatic stress.

	Terminated pregnancy		Continued pregnancy	
	Women (*N* = 87)	Men (*N* = 72)	Women (*N* = 93)	Men(*N* = 81)
	Mean (SD)	Mean (SD)	Mean (SD)	Mean (SD)
Time 1				
Depression	13.38 (5.47)	8.90 (4.87)	11.17 (6.10)	6.02 (5.16)
Intrusion	25.33 (8.85)	20.49 (9.19)	22.86 (10.08)	15.17 (9.51)
Avoidance	11.76 (6.29)	11.10 (7.19)	10.45 (8.19)	7.47 (7.49)
Arousal	13.21 (7.11)	9.25 (6.02)	12.10 (8.03)	6.46 (5.88)
Time 2				
Depression	7.95 (5.31)	4.18 (4.44)	5.31 (5.23)	2.77 (3.74)
Intrusion	18.70 (10.65)	13.89 (9.50)	11.67 (9.90)	8.69 (8.45)
Avoidance	9.28 (6.74)	7.38 (6.94)	6.10 (7.91)	4.10 (6.53)
Arousal	9.67 (7.39)	6.01 (5.68)	5.80 (6.55)	2.77 (3.74)

*Note*: Mean scores and standard deviations (SDs) for depression, assessed using the Edinburgh Postnatal Depression Scale (EPDS), and traumatic stress, assessed using the Impact of Event Scale (IES, including intrusion, avoidance and arousal), among pregnant couples with a recently detected fetal anomaly shortly after the time of detection (Time 1) and at the 6‐week follow‐up (Time 2).

### 3.2 Between‐group comparison

**FIGURE 2:**
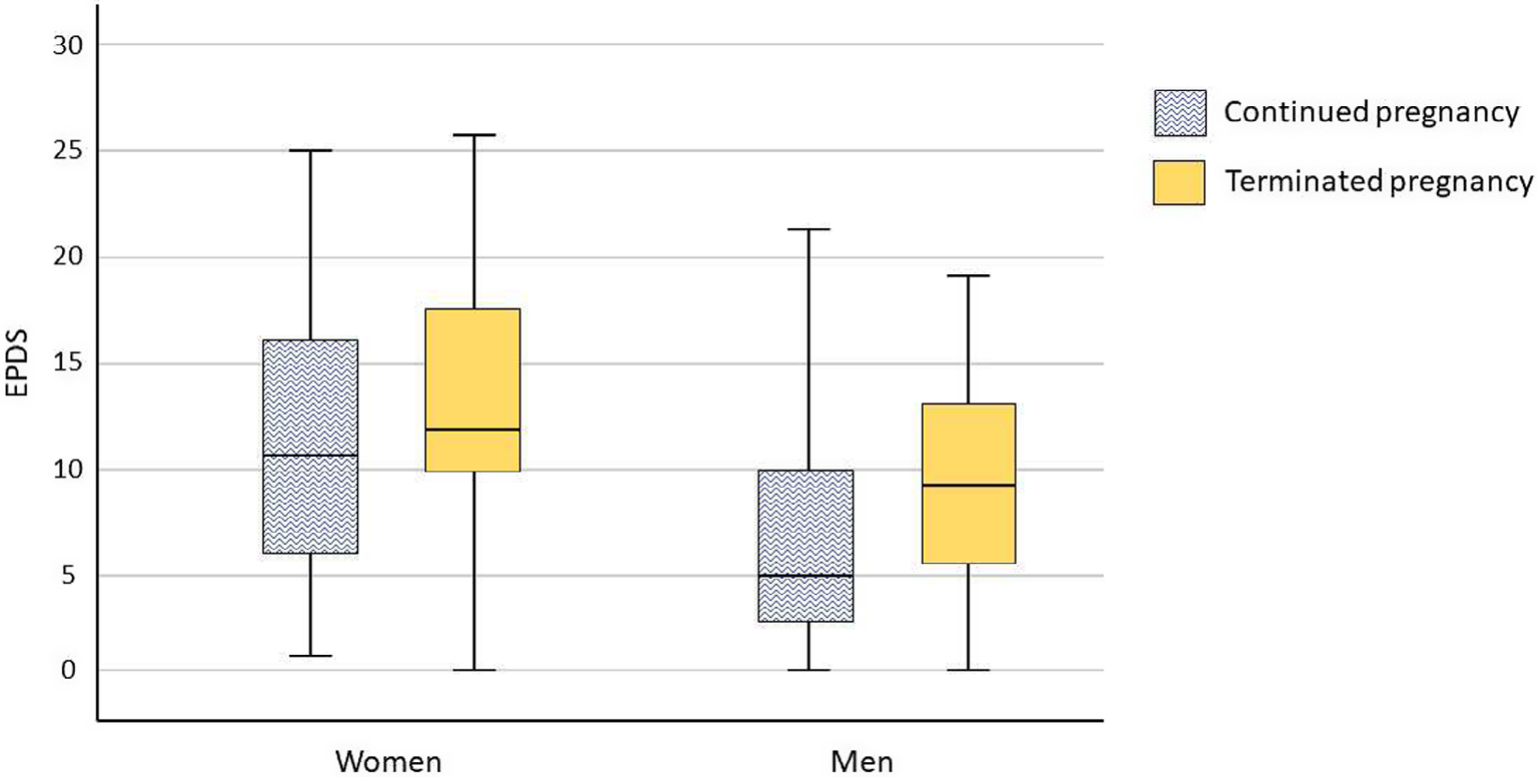
Depression, assessed with the Edinburgh Postnatal Depression Scale (EPDS), among pregnant couples with a recently detected fetal anomaly who later terminated (*n* = 87 women, *n* = 72 men) or did not terminate (*n* = 93 women, *n* = 81 men) the pregnancy.

Women who terminated the pregnancy reported more depressive symptoms prior to pregnancy termination than women who did not terminate the pregnancy after the detection of fetal anomalies, *t*(175) = 2.64, *P* = 0.009, mean difference 2.30 (95% CI 0.58–4.02) (Figure 2). Women who terminated the pregnancy did not report higher levels of intrusion, avoidance or arousal prior to termination than women who did not terminate the pregnancy.

Among men who experienced pregnancy termination, depression was significantly higher shortly after the detection than among men who continued the pregnancy, *t*(150) = 3.52, *P* < 0.001, mean difference 2.88 (95% CI 1.26–4.49) (Figure 2). Compared with men who did not terminate the pregnancy, men who experienced pregnancy termination also reported higher symptom levels on all subscales of traumatic stress prior to the termination: IES Intrusion, *t*(151) = 3.50, *P* < 0.001, mean difference 5.31 (95% CI 2.32–8.31); IES avoidance, *t*(151) = 3.50, *P* = 0.004, mean difference 3.63 (95% CI 1.18–6.07); and IES arousal, *t*(151) = 2.90, *P* = 0.004, mean difference 2.79 (95% CI 0.89–4.70) ( Figure S1).

### 3.3 Trajectories over time

**FIGURE 3:**
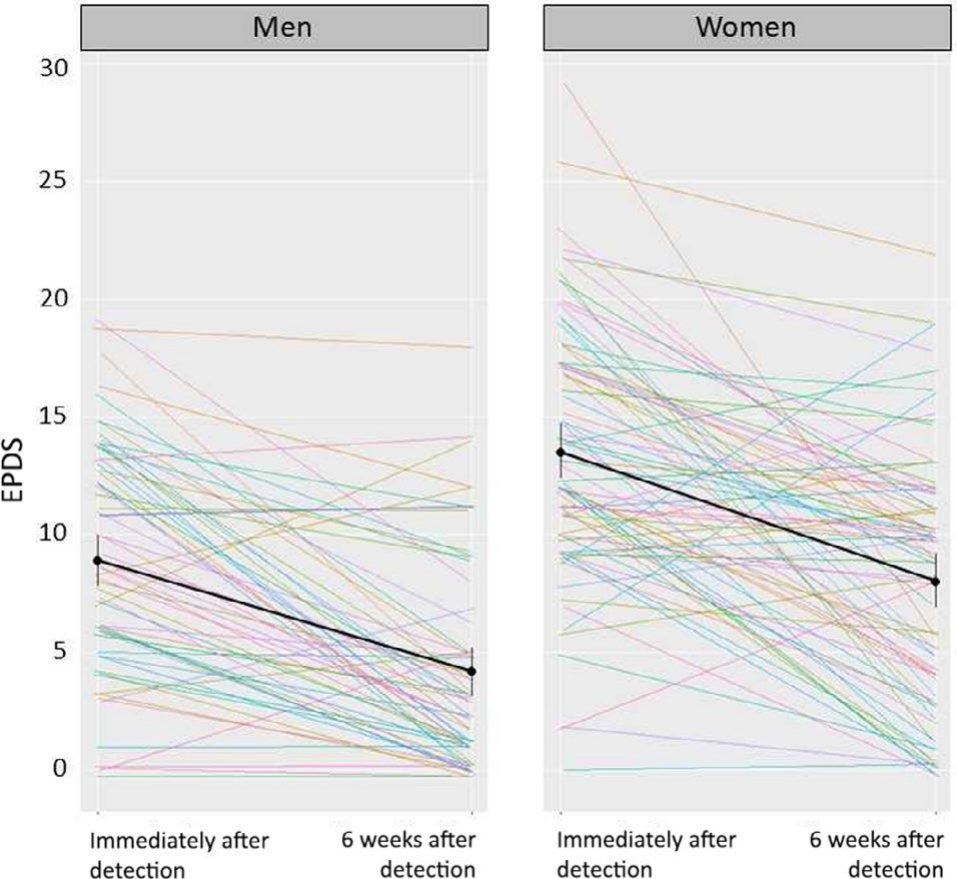
Depression, assessed with the Edinburgh Postnatal Depression Scale (EPDS), among men (*n* = 72) and women (*n* = 87) who terminated a pregnancy following the detection of fetal anomaly, immediately upon detection and at 6 weeks after pregnancy termination.

None of the potential confounding factors predicted depression or traumatic stress for women or men; therefore, no control variables were included in the models used to examine distress trajectories over time. In terms of depression, among expectant parents who terminated the pregnancy following the detection of a fetal anomaly, model 1 showed a significant main effect of time, such that depression decreased over time from before pregnancy termination to 6 weeks after termination (β = −5.18, SE = 0.55, χ^2^(1) = 73.50, *P* < 0.001) (Figure 3). There was also a main effect of gender, such that women experienced more depressive symptoms over time than men (β = 4.33, SE = 0.48, χ^2^(1) = 142.09, *P* < 0.001). There was no interaction between time and gender for depression.

Similarly, for all subscales of traumatic stress, symptoms decreased over time: IES Intrusion, β = −6.76, SE = 0.93, χ^2^(1) = 47.22, *P* < 0.001 (Figure S2); IES avoidance, β = −3.04, SE = 0.65, χ^2^(1) = 21.05, *P* < 0.001; and IES arousal, β = −3.47, SE = 0.64, χ^2^(1) = 26.98, *P* < 0.001. In terms of gender, women also experienced higher symptom levels than men over time: IES intrusion, β = 5.27, SE = 0.88, χ^2^(1) = 80.43, *P* < 0.001; IES avoidance, β = 1.42, SE = 0.65, χ^2^(1) = 25.75, *P* < 0.001; and IES arousal, β = 3.85, SE = 0.61, χ^2^(1) = 63.90, *P* < 0.001. There were no interaction effects between time and gender for any of the traumatic stress subscales.

## 4 DISCUSSION

### 4.1 Main findings

The present study showed that expectant parents who terminated the pregnancy following the detection of fetal anomalies reported significantly higher levels of depression prior to pregnancy termination than pregnant couples who did not terminate the pregnancy. Among men, but not among women, there was also a difference in traumatic stress prior to termination. Over time, women who terminated the pregnancy experienced overall higher levels of depression and traumatic stress than men. However, for both women and men, these levels decreased at the 6‐week follow‐up, but symptoms remained significantly higher among women.

### 4.2 Strengths and limitations

A major strength of this study is the unique prospective observational data for both women and men after the detection of fetal anomalies following ultrasound examination. We were able to compare the psychological responses of women and men before and 6 weeks after the TOP. We also compared these responses in the acute phase with those who did not terminate. Unlike many other studies, ours included both sexes and achieved high response rates at both assessment points. Additionally, our study employed well‐known, validated assessment methods to assess psychological reactions to trauma. However, one limitation of the current study is that we did not gather follow‐up data later than 6 weeks post‐termination. Further, it is possible that the difference in depression among men prior to pregnancy termination and men who did not terminate the pregnancy could be associated with other factors not measured in our study. Of note, some couples may make decisions about pregnancy termination during their ultrasound examination, and thus although the time of data collection happens before pregnancy termination, we cannot say with certainty that the decision has not already been made by the expectant parents. The observed decrease in symptoms among those who terminated might also be attributed to the timing of the 6‐week follow‐up; thus, future studies should preferably include follow‐up data closely after TOP and during a longer follow‐up period.

### 4.3 Interpretation

One interpretation could be that making the decision to terminate a pregnancy following the detection of fetal anomalies could influence depression and traumatic stress, and thus may have caused higher levels of distress prior to termination than among couples who did not terminate the pregnancy.^23^ For example, a qualitative study of 107 women and men who had terminated a pregnancy for fetal anomalies found that both partners retrospectively identified making the decision to terminate the pregnancy as the hardest part of the process.^24^ Indeed, our research shows that for women and men who terminated the pregnancy, feelings of depression and traumatic stress were strongest prior to termination. Our findings underscore significant gender differences in depression and traumatic stress responses to the detection of fetal anomalies and subsequent pregnancy termination. Initially, women who terminated the pregnancy exhibited higher depressive symptoms than their male partner, with scores surpassing the clinical depression threshold. This suggests that women may experience more intense immediate psychological distress following the detection of fetal anomalies. Previous research has found that both women and men are likely to see the experience of pregnancy termination as a trauma rather than a loss event. The finding that expectant men show less severe distress than women is consistent with previous research.^25^ However, the trajectories over time were similar between men and women, with both showing comparable declines in distress over time. Some previous research has speculated that women and men react to pregnancy termination for fetal anomalies very differently, with men being more likely to express a more limited immediate emotional response to protect their partner in a time of crisis, and subsequently being more likely to experience a delayed emotional reaction.^26^ Additionally, our data reveal that women consistently reported higher levels of all subcomponents of traumatic stress (intrusion, avoidance and arousal) compared with men throughout the study period. The persistent higher levels of traumatic stress indicators in women might reflect the direct emotional burden carried by expectant mothers facing fetal anomalies and the decisions surrounding them. These gender‐specific patterns of psychological response highlight the necessity for targeted support interventions that consider these differences.

### 4.4 Implications for practice

In the present as well as other studies, the detection of fetal anomalies continues to be an extremely distressing event for pregnant couples. Shock and distress may limit the ability of people to process information and deliberate about further options.^27^ It is therefore vital to consider how extreme emotional responses to prenatal diagnosis may impact an individual's capacity to process information and make informed choices.

This study highlights the importance of recognising the significant psychological distress involved, particularly prior to the decision‐making process, for both partners. Indeed, previous qualitative research has found that the time from the diagnosis of fetal anomaly to the time of termination is important, but that the emotional impact of the diagnosis can be lessened when good care is delivered.^10^ Thus, the time and duration from detecting an anomaly to pregnancy termination are crucial factors influencing mental health, making our findings highly relevant for clinical practice.

Our findings also highlight the different ways in which men and women respond to the stress of a pregnancy affected by fetal anomalies, thus, suggesting a need for professionals to offer counseling that adress the specific needs of men and women. Indeed, in a recent qualitative study examining the experience of fetal medicine specialists, the complex needs for care in women during this process was highlighted.^28^ In addition, fetal medicine specialists address the importance of being able to provide full care for women. Our findings further suggest that women might benefit from immediate, intensive support post‐diagnosis, focusing on emotional processing and validation. In the qualitative study by Power and colleagues, the care for men was not addressed.^28^ For men, continuing support might be especially beneficial, helping them to gradually work through their feelings over time. Understanding the shared and unique experiences of women and men during this period can guide practitioners in providing clear communication, non‐judgmental support and timely access to counselling services.

## 5 CONCLUSION

Overall, our study indicates that the levels of depression and traumatic stress peak before the TOP, specifically in the period following the detection of a fetal anomaly. Moreover, the follow‐up measures indicate a decrease in these symptoms 6 weeks after the termination. This implies that the decision‐making process unfolds during an intensely stressful time, suggesting that the severity of the fetal anomaly might affect prospective parents’ mental health more profoundly than the actual termination does. Although there were discernible differences in the levels of depression and traumatic stress between women and men, it is crucial to highlight that men also report significant traumatic stress. This is especially true during the decision‐making period. Notably, our research provides new insights into the experiences of fathers in this context, an often overlooked aspect in previous studies.

## AUTHOR CONTRIBUTIONS

AK and GH contributed to the design and implementation of the study. AK and GH have long clinical practice within Fetal Medicine. AK, GH, MB and AO conceptualised and planned the study. MB and AO wrote the article, with support from GH, BM and AK. AO conducted the statistical analyses, with contributions from all authors. MB, GH and AK contributed to funding acquisition.

## FUNDING INFORMATION

This work was supported by the Research Council of Norway (RCN, grants 288083 and 301004) and the Norwegian Women's Public Health Association.

## CONFLICT OF INTEREST STATEMENT

The authors have no conflicts of interest to declare.

## Supporting information


Figure S1.
Figure S2.
Table S1.

## Data Availability

The data that support the findings of this study are available on request from the corresponding author. The data are not publicly available owing to privacy or ethical restrictions.
