# Supplementary material for: Acute symptoms of depression and traumatic stress in men and women who terminate pregnancy after the detection of fetal anomaly: A prospective observational study
Source: BJOG. 2024 Jun 20;133(8):1539–46. doi: 10.1111/1471-0528.17884 (PMC13254023; doi:10.1111/1471-0528.17884)
Supplement: Supplementary file 1 [file BJO-133-1539-s001.odt]

Supplement 1

A chart with a number of boxes

Description automatically generated with medium confidence

Figure S1. Impact of Even Scale (IES) subscale Intrusion among pregnant couples with a recently detected fetal anomaly who later terminated (n=87 women, n=72 men) or did not terminate the pregnancy (n=93 women, n=81 men). 

Figure S2. Impact of Event Scale (IES) subscale Intrusion among men (n=72) and women (n=87) who terminated a pregnancy following the detection of fetal anomaly from acutely after detection until six weeks after pregnancy termination. 

Table S1. Psychological distress among mothers and fathers who terminated and did not terminate pregnancy following the detection of fetal anomaly. 

|  | Mothers (n=180) | Fathers (n=153) |
| --- | --- | --- |
|  | Terminated pregnancy M (SD) | Continued pregnancy M (SD) | p-value | Terminated pregnancy M (SD) | Continued pregnancy M (SD) | p-value |
| Acutely after detection | N=87 | N=93 |  | N=72 | N=81 |  |
| EPDS Total | 13.38 (5.47) | 11.17 (6.10) | .006 | 8.90 (4.87) | 6.02 (5.16) | <.001 |
| IES Intrusion | 25.33 (8.85) | 22.86 (10.08) | .085 | 20.49 (9.19) | 15.17 (9.51) | <.001 |
| IES Avoidance | 11.76 (6.20) | 10.45 (8.19) | .234 | 11.10 (7.82) | 7.47 (7.49) | .004 |
| IES Arousal | 13.21 (7.11) | 12.10 (8.03) | .329 | 9.25 (6.01) | 6.46 (5.88) | .004 |
| Six weeks after termination | N=83 |  |  | N=71 |  |  |
| EPDS Total | 7.95 (5.31) | - |  | 4.18 (4.44) | - | <.001 |
| IES Intrusion | 18.70 (10.65) | - |  | 13.89 (9.50) | - | .003 |
| IES Avoidance | 9.28 (6.74) | - |  | 7.38 (6.94) | - | .038 |
| IES Arousal | 9.67 (7.39) | - |  | 6.01 (5.68) | - | .010 |
